# Supplementary material for: Metabolic capabilities mute positive response to direct and indirect impacts of warming throughout the soil profile
Source: Nat Commun. 2021 Apr 7;12:2089. doi: 10.1038/s41467-021-22408-5 (PMC8027381; doi:10.1038/s41467-021-22408-5)
Supplement: Supplementary file 1 — Supplementary Information [file 41467_2021_22408_MOESM1_ESM.pdf]

Supplementary Information for "Metabolic capabilities mute positive response to direct and indirect impacts of warming throughout the soil profile."

Nicholas C. Dove[1,2*], Margaret S. Torn[3], Stephen C. Hart[4], Neslihan Taş[3,5*]

[1]Environmental Systems Graduate Group, University of California, Merced, CA 95343, USA

[2]Biosciences Division, Oak Ridge National Laboratory, Oak Ridge, TN 37830, USA

[3]Earth and Environmental Sciences Area, Lawrence Berkeley National Laboratory, Berkeley, CA 94720, USA

[4]Department of Life & Environmental Sciences and Sierra Nevada Research Institute, University of California, Merced, CA 95343, USA

[5]Biosciences Area, Lawrence Berkeley National Laboratory, Berkeley, CA 94720, USA

*Corresponding author: Neslihan Taş (ntas@lbl.gov) or Nicholas C. Dove (ndove7@gmail.com); phone: +1-510-486-5538

SUPPLEMENTARY METHODS

The field experiment is first explained in Hicks Pries et al.[1]. The heating treatment warmed the soil 4 °C above ambient temperatures to 1 m depth while maintaining the natural temperature gradient with depth following the design of Hanson et al.[2]. The experimental design consisted of three paired heated plots and unheated control plots (circular, 3 m in diameter). Twenty-two 2.4 m long steel pipes surrounded each plot, at 0.25 m beyond the plot perimeter to mitigate potential hot spots near the heaters. In heated plots, resistance heater cable (BriskHeat, Ohio, USA) was placed inside the pipes, which were then filled with sand. Steel pipes around the control plots contained sand only. Two concentric rings of heater cable at 1 and 2 m in diameter, 5 cm below the soil surface, were installed to compensate for surface heat loss. Unheated cables were installed similarly in control plots. Power to the heaters was routed through silicon-controlled rectifiers (SCRs, Watlow, Missouri, USA) controlled by a CR1000 datalogger (Campbell Scientific, Utah, USA) through a current-to-voltage converter (SDM-CVO4, Campbell Scientific, Utah, USA). The amount of power supplied to the deep heaters was based on the temperature difference between paired control and heated thermistors at 75 and 100 cm depth at a radial distance of 0.75 m from plot center. Power supplied to the surface heaters was based on the temperature difference of thermistors at 15 and 20 cm depth at a radial distance of 0 and 0.75 m from plot center.

SUPPLEMENTARY FIGURES

Supplementary Figure 1: Boxplots (n = 3) representing microbial respiration rates measured periodically across two depths amended with cellobiose (+C) and cellobiose, nitrogen, and phosphorus (+CNP) over the 30-d incubation. For boxplots, the median is indicated by the thick black line, and the lower and upper hinges correspond to the first and third quartiles. The lower and upper whiskers extend to smallest or largest value, respectively, no further than 1.5 times the interquartile range.

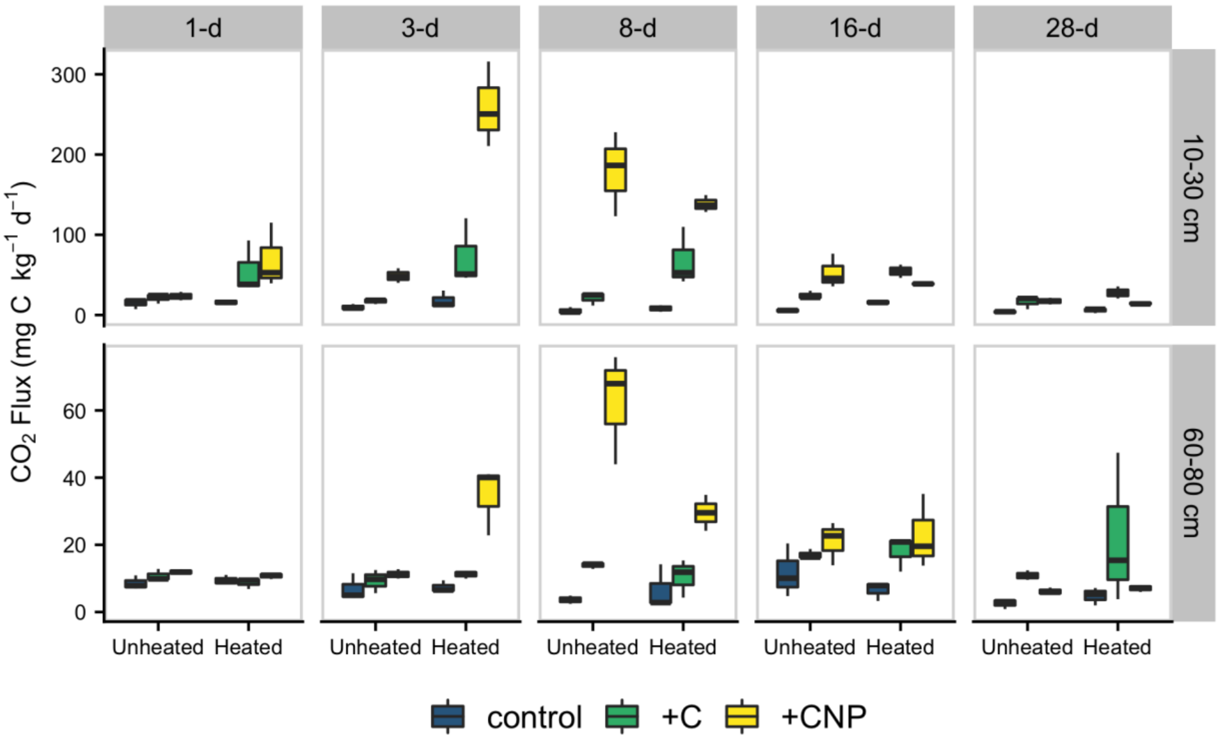

Supplementary Figure 2: Relative abundance of ten most abundant fungal families across heating treatments throughout the soil profile.

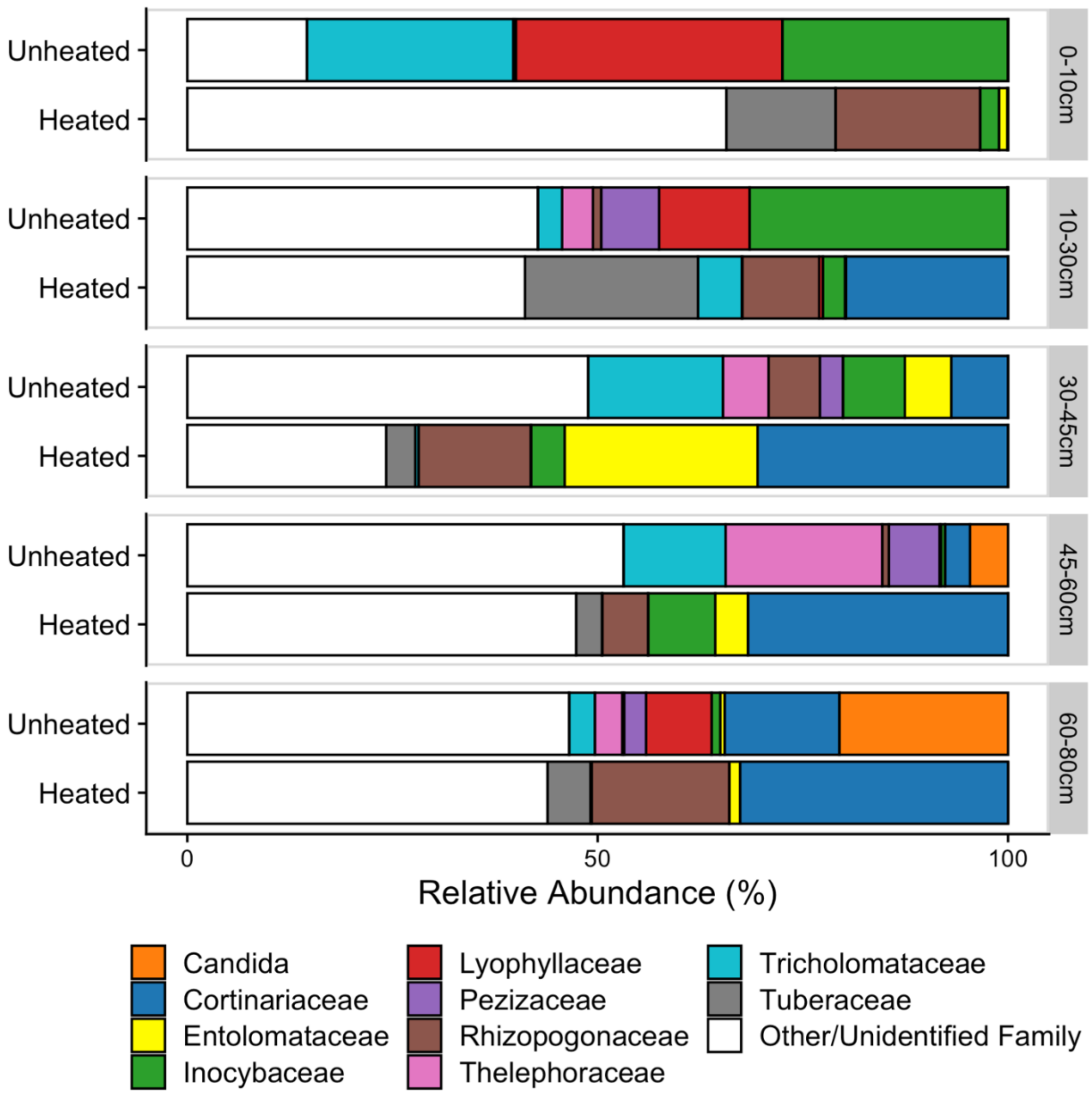

Supplementary Figure 3: Mean relative abundance of ectomycorrhizal (EM) fungal reads are shown for heated (red) and unheated (gray) soils. Error bars show ± one standard error (n = 3).

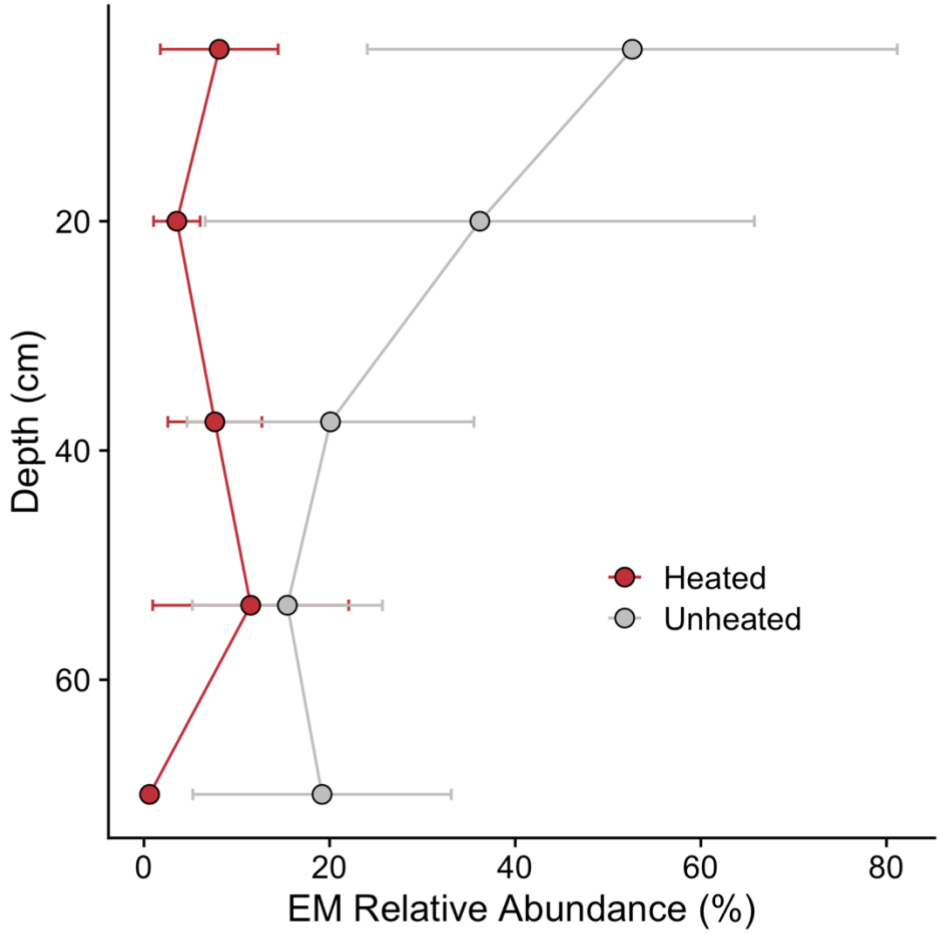

Supplementary Figure 4: Relative abundance of major bacterial phyla across heating treatments throughout the soil profile by 16S (A) and shotgun metagenomics (B).

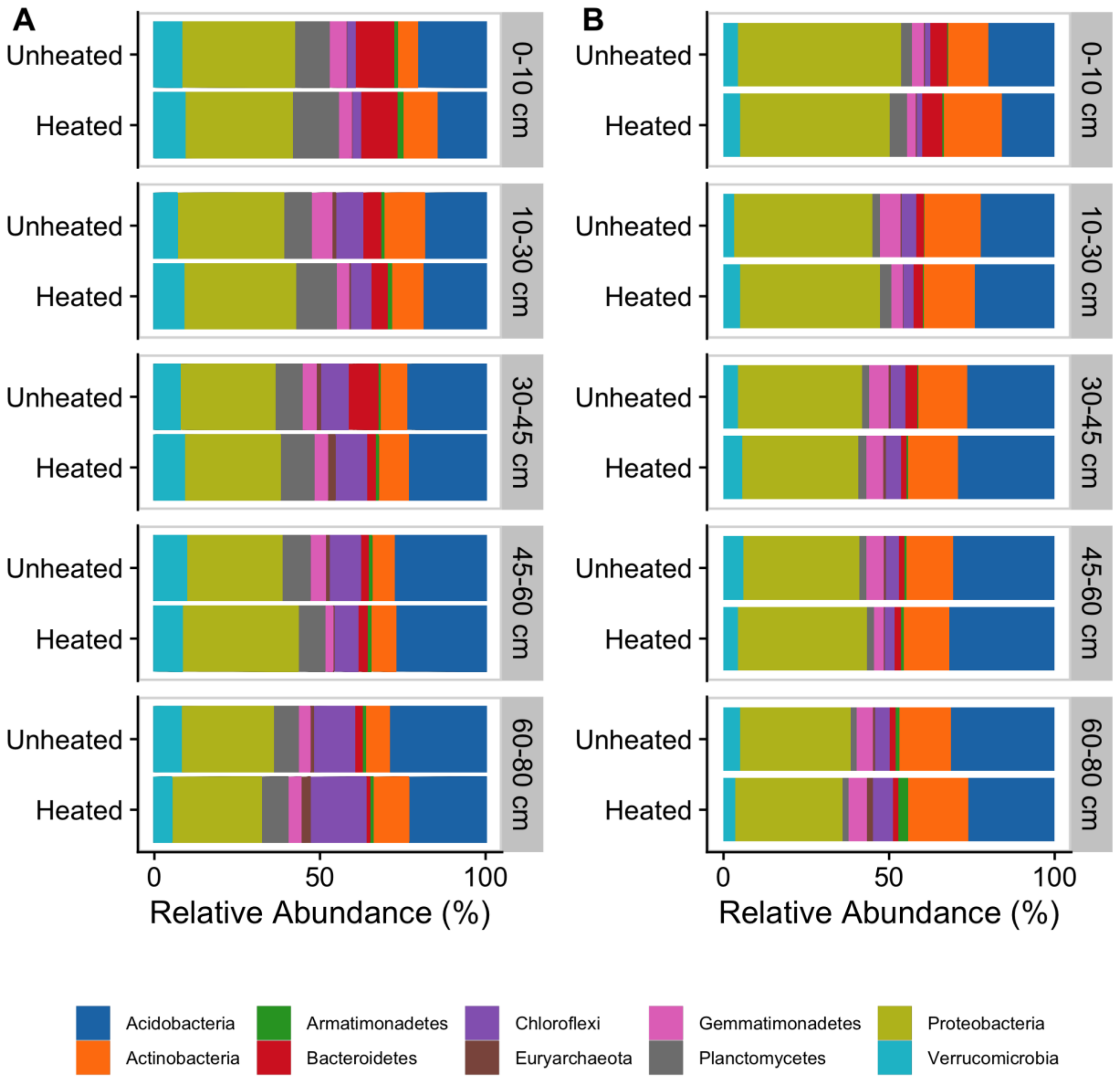

Supplementary Figure 5: Mean Chao1 (A) and Shannon (B) index of the archaeal & bacterial (Archaea/Bacteria) and fungal communities are shown for heated (red) and unheated (gray) soils. Error bars show ± one standard error (n = 3).

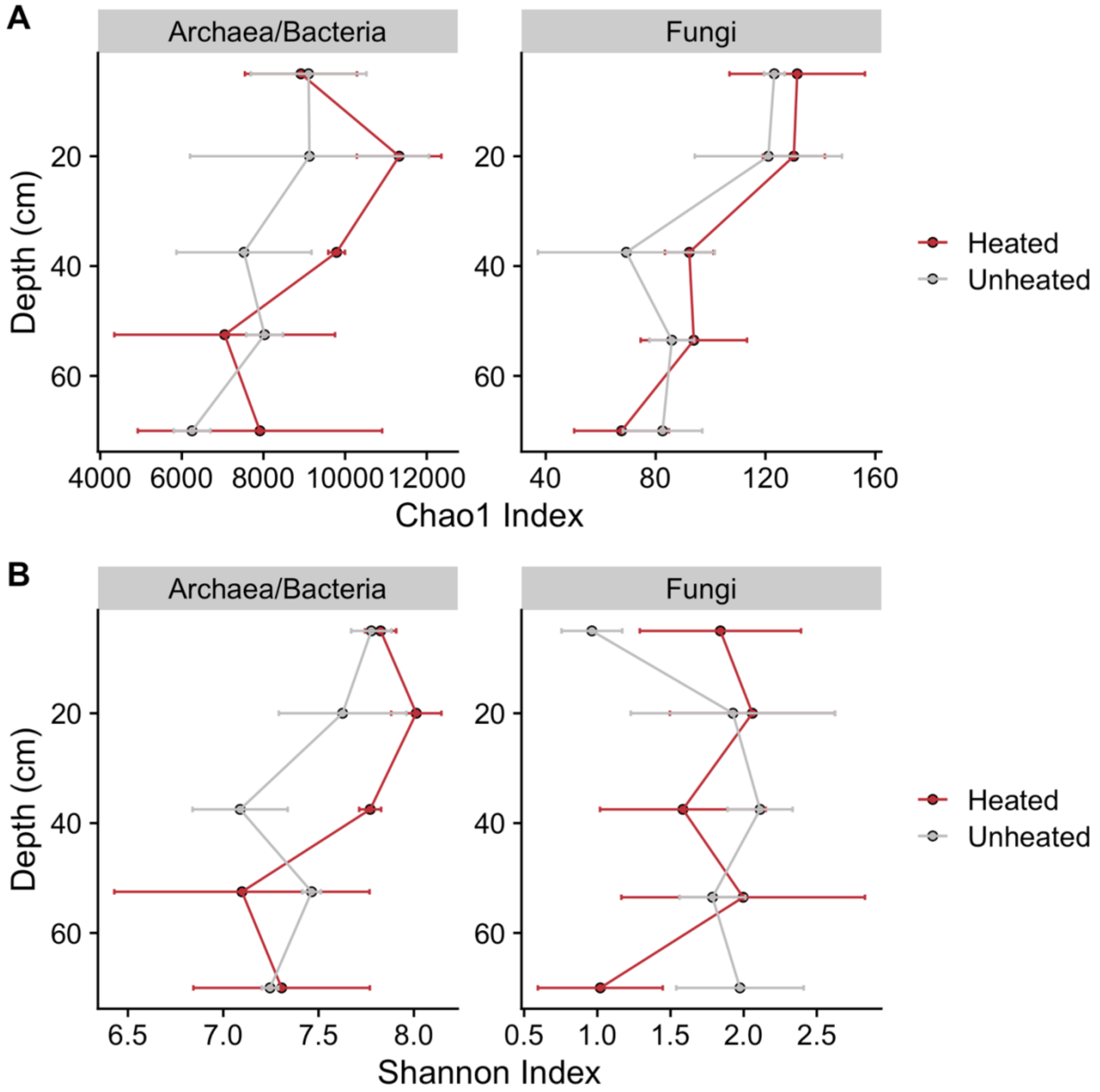

Supplementary Figure 6: Relative abundance of major bacterial phyla across heating treatments throughout the soil profile over a 30-d incubation amended with carbon (C) or carbon, nitrogen, and phosphorus (CNP) compared to the starting conditions and unamended control.

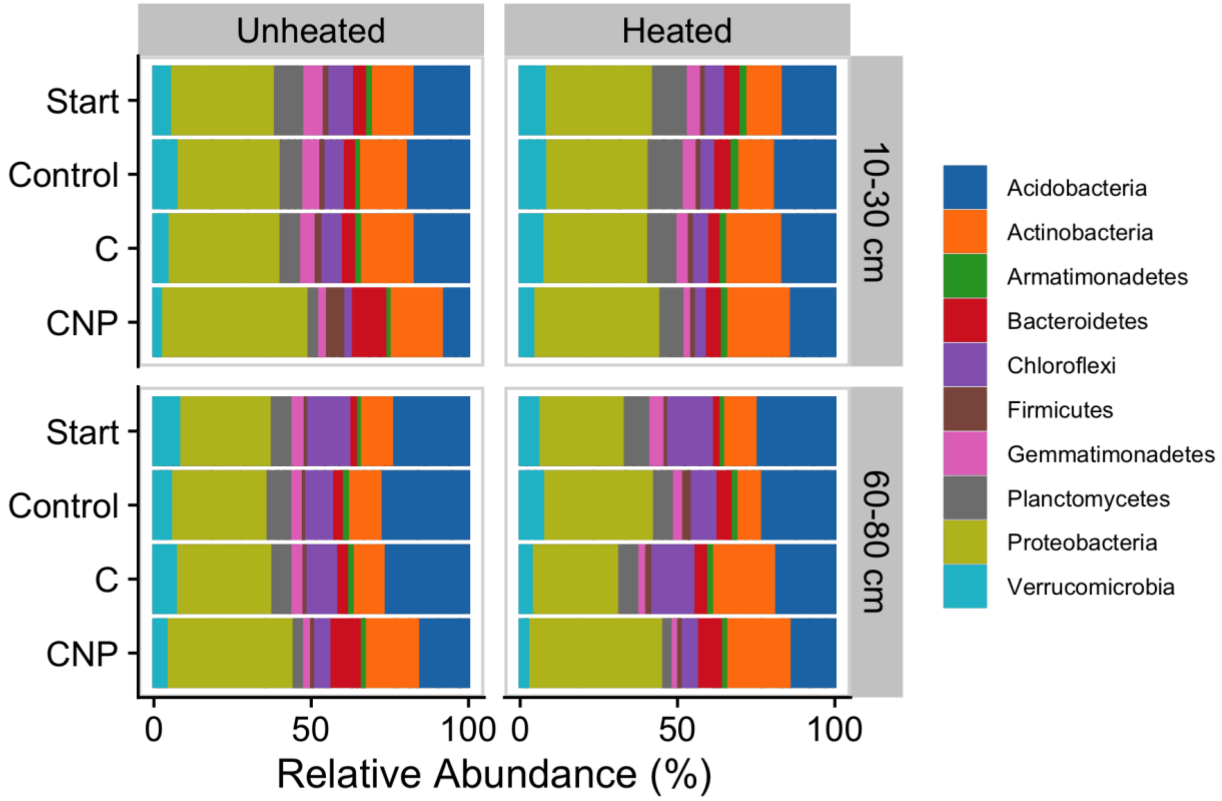

Supplementary Figure 7: Boxplots (n = 3) showing resource limitations of prokaryote (bacteria and archaea) community composition shift (assessed by Bray-Curtis distance) across heating treatments. Carbon (C) limitation is assessed by the response of the C-amended soils divided by the control soil, and nutrient limitation is assessed by the response of the C- and nutrient-amended soil divided by the C-amended soil. Dashed line at 1.0 indicates no limitation. For boxplots, the median is indicated by the thick black line, and the lower and upper hinges correspond to the first and third quartiles. The lower and upper whiskers extend to smallest or largest value, respectively, no further than 1.5 times the interquartile range.

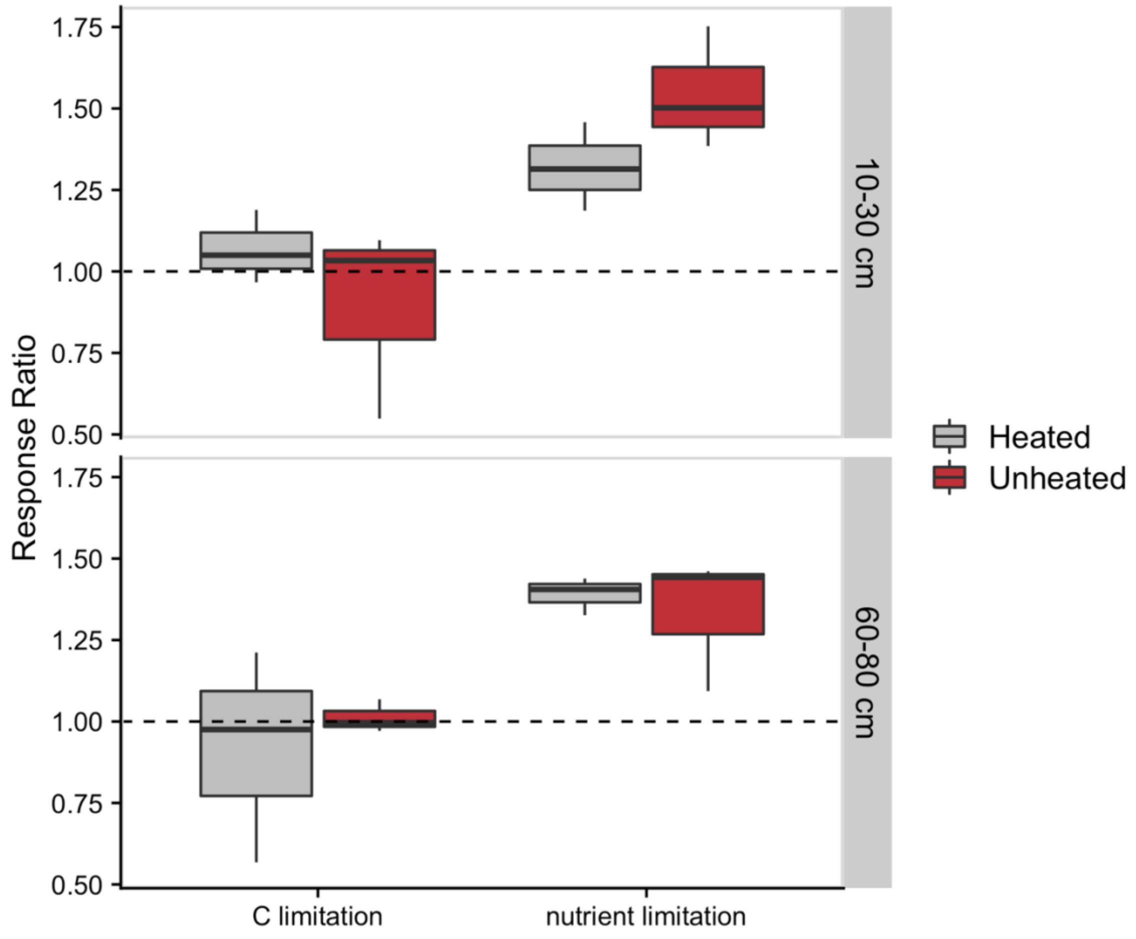

Supplementary Figure 8: Average microbial functional genes abundance normalized by amino acid (AA) coding reads for each depth across heated and unheated plots. Error bars show ± one standard error (n = 3).

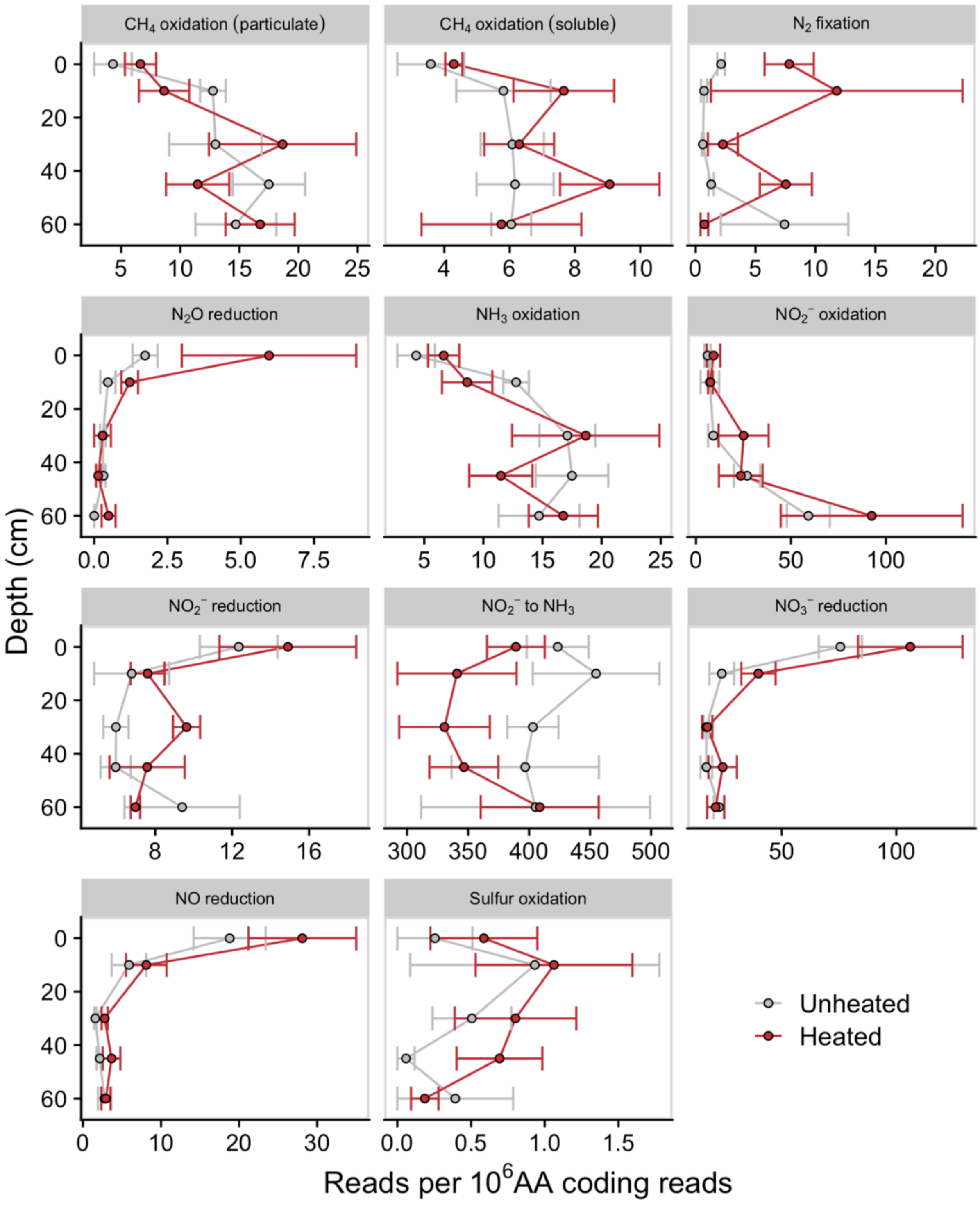

Supplementary Figure 9: Relative abundance of metagenome assembled genomes (MAGs) across heating treatments and throughout the soil profile.

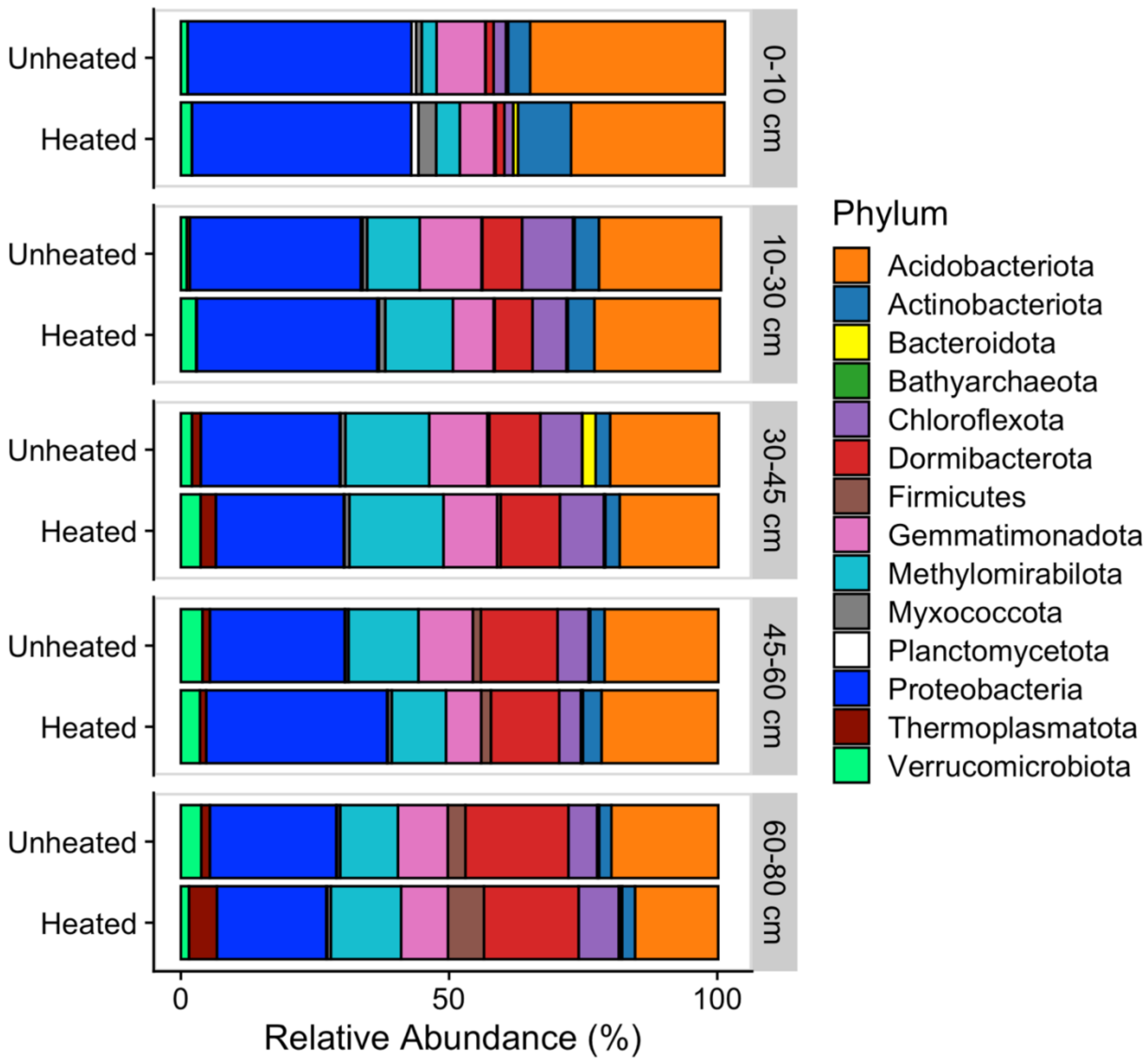

Supplementary Figure 10: Estimated metagenome assembled genome (MAG) growth rates across heating treatments and depths. For boxplots, the median is indicated by the thick black line, and the lower and upper hinges correspond to the first and third quartiles. The lower and upper whiskers extend to smallest or largest value, respectively, no further than 1.5 times the interquartile range.

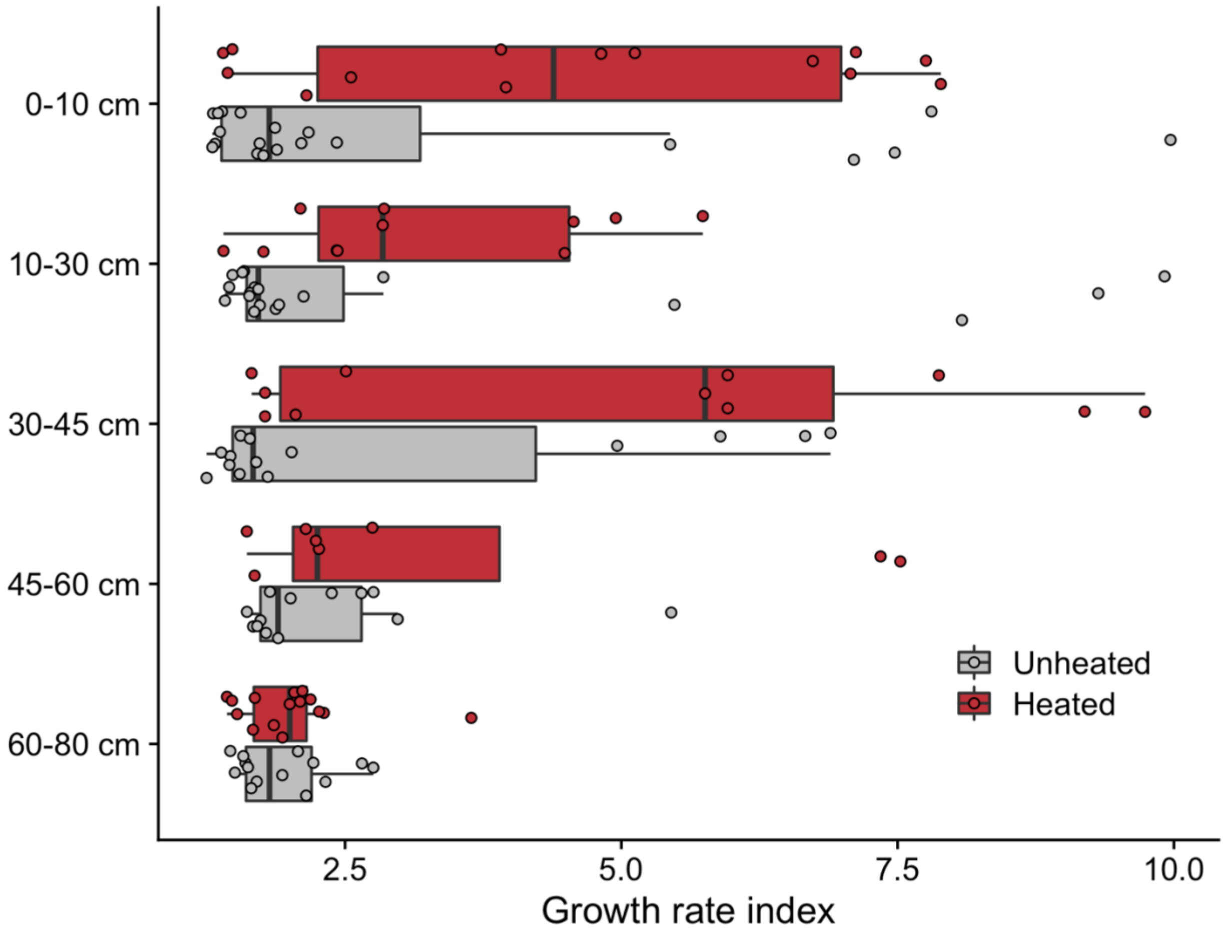

Supplementary Figure 11: Metabolic pathways with genes (box) annotated to a known function display a variety of metabolic functions in *Actinobacteria* (*Acidimicrobiia*) MAG that showed the highest increase in its estimated growth rate under warming. Cell morphology is arbitrarily displayed. Pathways; PPP: Pentose Phosphate Pathway (non-oxidative phase), TCA: Citric Acid Cycle (incomplete), Gly: Glycolysis – Glyconeogenesis, PRPP: Phosphoribosyl pyrophosphate biosynthesis, TAT: The twin-arginine translocation protein export system, Type II FAS: Type II fatty acid synthesis pathway, AS reduction: Arsenate reductase with glutaredoxin (EC 1.20.4.1) and thioredoxin (EC1.20.4.4), CAZymes; GT2: Glycoside transferase 2, Genes; GS: Glutamine synthetase (EC 6.3.1.2), GOGDP: Glutamate synthetase (EC 1.4.1.13), Urease: Urease (EC:3.5.1.5), SDH: succinate dehydrogenase (EC 1.3.5.1), FDH: Formate dehydrogenase (EC 1.2.1.46), XYL: Beta-xylosidase (EC 3.2.1.37), AMY: Amylase (EC 3.2.1.1), MANA: Mannose-6-phosphate isomerase (EC 5.3.1.8), MANB: Beta-mannosidase (EC 3.2.1.25), CYC-C: Cytochrome C oxidase cbb3-type (EC 7.1.1.9), PHOD: Alkaline phosphatase D (EC 3.1.3.1), PPA: Inorganic pyrophosphatase (EC 3.6.1.1), NUO: NADH-quinone oxidoreductase subunit A (EC 7.1.1.2), COX: Aerobic carbon-monoxide dehydrogenase (EC 1.2.5.3).

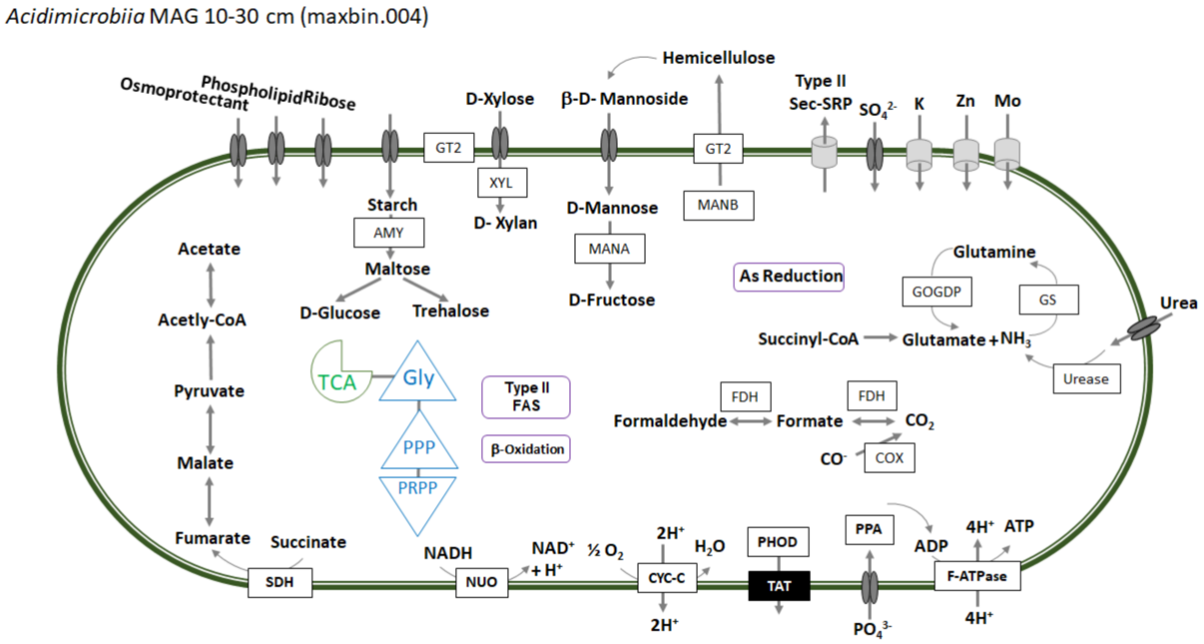

SUPPLEMENTARY TABLES

Supplementary Table 1: Mean (and standard error) microbial biomass carbon (MBC) among depths and across heating treatments (n = 6).

Depth (cm)	MBC (mg kg^{-1})
10-20	186.7 (78.3)
50-60	48.8 (10.5)
70-80	27.1 (20.3)

Supplementary Table 2: Mean (and standard error of the mean, n = 3) diversity (D_{NP}) estimates of metagenomes from Nonpareil[1].

Depth (cm)	Unheated	Heated
0-10	23.11 (0.13)	22.86 (0.04)
10-30	22.50 (0.26)	22.79 (0.10)
30-45	22.25 (0.27)	22.56 (0.02)
45-60	22.23 (0.10)	22.56 (0.20)
60-80	21.99 (0.09)	21.88 (0.37)

Supplementary Table 3: Average soil total carbon and nitrogen (N) concentrations, net N mineralization (assessed during a 30-d laboratory incubation), and gravimetric water content (GWC) across heating treatments throughout the soil profile, with standard error of the mean (n = 3) in parentheses. Net N mineralization was assayed only in the 10-30 and 60-80 cm horizons.

	Carbon (%)		Nitrogen (%)		net N mineralization (mg kg^{-1} 30-d^{-1})		GWC (g H_2O g^{-1} dry soil)	
Depth	unheated	heated	unheated	unheated	unheated	heated	unheated	heated
0-10 cm	10.9 (0.893)	8.06 (0.23)	0.251 (0.031)	-	-	-	-	-
10-30 cm	2.65 (0.563)	3.15 (0.312)	0.101 (0.021)	0.72 (0.21)	0.72 (0.21)	5.73 (2.41)	0.21 (0.01)	0.16 (0.05)
30-45 cm	1.07 (0.070)	1.46 (0.321)	0.034 (0.002)	-	-	-	-	-
45-60 cm	0.620 (0.073)	0.605 (0.009)	0.023 (0.001)	-	-	-	-	-
60-80 cm	0.458 (0.146)	0.396 (0.005)	0.016 (0.003)	0.33 (0.28)	0.33 (0.28)	0.97 (0.47)	0.16 (0.01)	0.14 (0.02)

Supplementary Table 4: Summary statistics for correlations (Spearman's *Rho*, two-sided) between metagenome associated metagenome (MAG) relative abundance and depth across heating treatments shown for each phyla (n = 6). Significant ($p < 0.050$) correlations are bolded. P-values are corrected using the false discovery rate correction[3].

Phylum	p-value	*Rho*
Acidobacteriota	0.095	-0.666
**Actinobacteriota**	**0.001**	**-0.840**
Bacteroidota	0.335	-0.578
Chloroflexota	1.000	0.284
**Bathyarchaeota**	**< 0.001**	**0.867**
**Dormibacterota**	**< 0.001**	**0.906**
**Firmicutes**	**< 0.001**	**0.906**
Gemmatimonadota	1.000	0.087
Methylomirabilota	0.883	0.491
**Myxococcota**	**0.010**	**-0.775**
Planctomycetota	0.496	-0.546
**Proteobacteria**	**0.035**	**-0.720**
**Thermoplasmatota**	**0.010**	**0.775**
Verrucomicrobiota	0.631	0.524

Supplementary Table 5: Primer sequences for polymerase chain reaction (PCR) amplification.

Primer Name	Sequence	Direction
515F	GTGCCAGCMGCCGCGGTAA	Forward
806R	GGACTACHVGGGTWTCTAAT	Reverse
ITS1f	CTTGGTCATTTAGAGGAAGTAA	Forward
ITS2	GCTGCGTTCTTCATCGATGC	Reverse

Supplementary Table 6: Coverage and Diversity (D_{NP}) estimates of metagenomes from Nonpareil[4].

Plot	Heating Treatment	Depth	Coverage (%)	LR[a]	LR*[b]	D_{NP}
1	Unheated	0-10 cm	42.79	1.07E+10	4.59E+11	23.33
1	Unheated	10-30 cm	54.46	9.93E+09	3.32E+11	22.61
1	Unheated	30-45 cm	63.04	1.09E+10	2.21E+11	22.16
1	Unheated	45-60 cm	57.84	9.25E+09	2.51E+11	22.31
1	Unheated	60-80 cm	58.53	6.75E+09	1.79E+11	21.94
1	Heated	0-10 cm	41.30	6.55E+09	3.71E+11	22.92
1	Heated	10-30 cm	49.76	7.30E+09	2.34E+11	22.59
1	Heated	30-45 cm	47.56	6.13E+09	2.42E+11	22.52
1	Heated	45-60 cm	42.48	6.68E+09	3.28E+11	22.88
1	Heated	60-80 cm	56.17	5.72E+09	1.57E+11	21.99
2	Unheated	0-10 cm	51.76	1.41E+10	6.99E+11	23.12
2	Unheated	10-30 cm	48.13	9.13E+09	4.63E+11	22.88
2	Unheated	30-45 cm	57.12	1.35E+10	3.70E+11	22.75
2	Unheated	45-60 cm	68.73	1.26E+10	1.70E+11	22.04
2	Unheated	60-80 cm	57.94	5.55E+09	1.20E+11	21.88
2	Heated	0-10 cm	43.70	6.47E+09	2.35E+11	22.78
2	Heated	10-30 cm	35.80	4.89E+09	3.51E+11	22.93
2	Heated	30-45 cm	50.60	7.64E+09	2.12E+11	22.60
2	Heated	45-60 cm	49.94	5.04E+09	1.81E+11	22.19
2	Heated	60-80 cm	70.67	7.21E+09	1.14E+11	21.20
3	Unheated	0-10 cm	44.14	7.17E+09	4.70E+11	22.87
3	Unheated	10-30 cm	55.83	6.08E+09	2.10E+11	22.00
3	Unheated	30-45 cm	57.01	5.16E+09	1.38E+11	21.83
3	Unheated	45-60 cm	56.68	8.31E+09	2.12E+11	22.34
3	Unheated	60-80 cm	50.80	5.16E+09	1.87E+11	22.16
3	Heated	0-10 cm	45.80	7.83E+09	2.59E+11	22.87
3	Heated	10-30 cm	40.64	5.99E+09	2.97E+11	22.85
3	Heated	30-45 cm	53.97	9.01E+09	2.58E+11	22.55
3	Heated	45-60 cm	55.27	1.01E+10	2.86E+11	22.60
3	Heated	60-80 cm	59.28	1.17E+10	3.36E+11	22.46

[a]Sequencing effort (bases)

[b]Sequencing effort required for nearly complete coverage

Supplementary Table 7: Comparison of carbon (C), nitrogen, (N), and phosphorus (P) additions in multiple resource amendment incubations. Dashes represent no addition of element.

Reference	C (g C kg^{-1} soil)	N (g N kg^{-1} soil)	P (g P kg^{-1} soil)	ecosystem
**This Study (topsoils)**	**3**	**0.35**	**0.10**	**Temperate forest**
**This Study (subsoils)**	**0.7**	**0.081**	**0.023**	**Temperate forest**
Reed et al.[5]	4	0.8	0.8	Tropical forest
Marañon-Jimenez et al.[6]	0.69	0.035	0.018	Temperate grassland
Soong et al.[7]	-	0.37	0.195	Tropical forest
Spiers and McGill[8]	10	0.88	0.5	Agricultural
Waldrop and Firestone[9]	-	0.03	-	Tropical forest
Ouyang et al.[10]	-	1.00-0.04	0.1	Temperate steppe

SUPPLEMENTARY REFERENCES

1. Hicks Pries, C. E., Castanha, C., Porras, R. C. & Torn, M. S. The whole-soil carbon flux in response to warming. *Science* **355**, 1420–1423 (2017).
2. Hanson, P. J. *et al.* A method for experimental heating of intact soil profiles for application to climate change experiments. *Glob. Change Biol.* **17**, 1083–1096 (2011).
3. Benjamini, Y. & Hochberg, Y. Controlling the false discovery rate: A practical and powerful approach to multiple testing. *J. R. Stat.l Soc. Series B* **57**, 289–300 (1995).
4. Rodriguez-R, L. M. & Konstantinidis, K. T. Nonpareil: a redundancy-based approach to assess the level of coverage in metagenomic datasets. *Bioinformatics* **30**, 629–635 (2014).
5. Reed, S. C., Vitousek, P. M. & Cleveland, C. C. Are patterns in nutrient limitation belowground consistent with those aboveground: results from a 4 million year chronosequence. *Biogeochemistry* **106**, 323–336 (2011).

6. Marañón-Jiménez, S. *et al.* Geothermally warmed soils reveal persistent increases in the respiratory costs of soil microbes contributing to substantial C losses. *Biogeochemistry* **138**, 245–260 (2018).

7. Soong, J. L. *et al.* Soil microbial CNP and respiration responses to organic matter and nutrient additions: Evidence from a tropical soil incubation. *Soil Biol. Biochem.* **122**, 141–149 (2018).

8. Spiers, G. A. & McGill, W. B. Effects of phosphorus addition and energy supply on acid phosphatase production and activity in soils. *Soil Biol. Biochem.* **11**, 3–8 (1979).

9. Waldrop, M. P. & Firestone, M. K. Altered utilization patterns of young and old soil C by microorganisms caused by temperature shifts and N additions. *Biogeochemistry* **67**, 235–248 (2004).

10. Ouyang, X. *et al.* Effect of N and P addition on soil organic C potential mineralization in forest soils in South China. *J. Environ. Sci.* **20**, 1082–1089 (2008).
