# Supplementary material for: Metabolic capabilities mute positive response to direct and indirect impacts of warming throughout the soil profile
Source: Nat Commun. 2021 Apr 7;12:2089. doi: 10.1038/s41467-021-22408-5 (PMC8027381; doi:10.1038/s41467-021-22408-5)
Supplement: Supplementary file 3 — Description of Additional Supplementary Files [file 41467_2021_22408_MOESM3_ESM.pdf]

Description of Additional Supplementary Files

File Name: Supplementary Data 1

Description: Metabolic functions in metagenome assembled genomes in separate spreadsheet.

File Name: Supplementary Data 2

Description: Description of metagenome assembled genomes in separate spreadsheet.

File Name: Supplementary Data 3

Description: Metabolic functions in positive or negative heat-responding metagenome assembled genomes in separate spreadsheet.
