# Supplementary material for: Metabolic capabilities mute positive response to direct and indirect impacts of warming throughout the soil profile
Source: Nat Commun. 2021 Apr 7;12:2089. doi: 10.1038/s41467-021-22408-5 (PMC8027381; doi:10.1038/s41467-021-22408-5)
Supplement: Supplementary file 7 — Reporting Summary [file 41467_2021_22408_MOESM7_ESM.pdf]

nature research

Corresponding author(s): Neslihan Tas

Last updated by author(s): Feb 18, 2021

Reporting Summary

Nature Research wishes to improve the reproducibility of the work that we publish. This form provides structure for consistency and transparency in reporting. For further information on Nature Research policies, see our Editorial Policies and the Editorial Policy Checklist.

Statistics

For all statistical analyses, confirm that the following items are present in the figure legend, table legend, main text, or Methods section.

n/a	Confirmed	
☐	☒	The exact sample size (*n*) for each experimental group/condition, given as a discrete number and unit of measurement
☐	☒	A statement on whether measurements were taken from distinct samples or whether the same sample was measured repeatedly
☐	☒	The statistical test(s) used AND whether they are one- or two-sided<br>*Only common tests should be described solely by name; describe more complex techniques in the Methods section.*
☐	☒	A description of all covariates tested
☐	☒	A description of any assumptions or corrections, such as tests of normality and adjustment for multiple comparisons
☐	☒	A full description of the statistical parameters including central tendency (e.g. means) or other basic estimates (e.g. regression coefficient) AND variation (e.g. standard deviation) or associated estimates of uncertainty (e.g. confidence intervals)
☐	☒	For null hypothesis testing, the test statistic (e.g. *F*, *t*, *r*) with confidence intervals, effect sizes, degrees of freedom and *P* value noted<br>*Give P values as exact values whenever suitable.*
☒	☐	For Bayesian analysis, information on the choice of priors and Markov chain Monte Carlo settings
☒	☐	For hierarchical and complex designs, identification of the appropriate level for tests and full reporting of outcomes
☐	☒	Estimates of effect sizes (e.g. Cohen's *d*, Pearson's *r*), indicating how they were calculated

Our web collection on statistics for biologists contains articles on many of the points above.

Software and code

Policy information about availability of computer code

Data collection	No software was used for data collection
Data analysis	Phylogenetic analysis was performed via QIIME version 1.7.0, UPARSE (v. 11) and MegaBlast. Mycorrhizal taxa were assigned using FUNGuild (v. 1.0). Raw sequence reads were trimmed and quality filtered using Trimmomatic (v. 0.36), and read taxonomy was classified using Kaiju (v. 1.6).Coverage and read diversity of each metagenome was calculated via Nonpareil (v. 3.3.3). Prodigal (v. 2.6.1) was used to predict coding regions from the reads. DIAMOND BLASTp (v. 0.8.36) was use to match against carbohydrate active enzymes using the CAZy database (CAZyDB.07312018). Nitrogen cycling, methane production and oxidation, and sulfate reduction genes were annotated via hmmer (v. 3.1b2) against KEGG database (downloaded 28-May-2019).<br><br>MEGAHIT (v. 1.1.3) and Metaspades (v. 3.13.0) were used to build contigs from raw reads. MaxBin (v. 2.2.5) and MetaBAT2 (v. 2.12.1), and DASTool (v. 1.1.10) was used to build genome bins. Potential misbinnings were identified with CheckM. GTDB-Tk (v. 0.3.2, database release 89) to classify each contig into a taxonomic rank from phylum to species. FastANI (v. 0.1.2) was used to compare metagenome assembled genomes (MAGs) across assemblies and for dereplication. Protein-encoding genes from MAGs were predicted with Prodigal (v. 2.6.1), . Contigs were annotated using DIAMOND BLASTX (v. 0.8.36) against KEGG database (downloaded 28-May-2019), RAST-Tk (v. 1.073 as implemented in KBase) and METABOLIC (v. 1.1). We estimated the growth rate of MAGs >75% completeness in each sample using GRiD (v 1.3).<br><br>All statistical analyses were conducted in R (v. 4.0.2) using the car, lme, phyloseq, DESeq2, and vegan packages.

For manuscripts utilizing custom algorithms or software that are central to the research but not yet described in published literature, software must be made available to editors and reviewers. We strongly encourage code deposition in a community repository (e.g. GitHub). See the Nature Research guidelines for submitting code & software for further information.

Data

Policy information about availability of data

All manuscripts must include a data availability statement. This statement should provide the following information, where applicable:

- Accession codes, unique identifiers, or web links for publicly available datasets
- A list of figures that have associated raw data
- A description of any restrictions on data availability

All sequence data are deposited at European Nucleotide Archive. 16S rRNA gene sequences can be found under the Bioproject PRJEB39495 (SAMEA7090473) and ITS gene sequences be found under the Bioproject PRJEB39496 (SAMEA7090474). The metagenome raw reads can also be found under the Bioproject PRJEB39497 (SAMEA7090475- SAMEA7090504), Metagenome assembled-genomes and their annotations available through KBase narrative: https://narrative.kbase.us/narrative/57664 (KBase account required). The following databases can be accessed using the following links: CAZy (http://www.cazy.org/) (CAZyDB.07312018), GTDB-Tk (v. 0.3.2) (https://github.com/Ecogenomics/GTDBTk), KEGG (https://www.kegg.jp/kegg/download/) (downloaded 28-May-2019), NCBI nr (https://www.ncbi.nlm.nih.gov/), SILVA (https://www.arb-silva.de/) (database 132).

Field-specific reporting

Please select the one below that is the best fit for your research. If you are not sure, read the appropriate sections before making your selection.

☐ Life sciences ☐ Behavioural & social sciences ☒ Ecological, evolutionary & environmental sciences

For a reference copy of the document with all sections, see nature.com/documents/nr-reporting-summary-flat.pdf

Ecological, evolutionary & environmental sciences study design

All studies must disclose on these points even when the disclosure is negative.

Study description	In order to determine the direct and indirect impacts of soil warming on microbial community composition, physiology, and metabolism throughout the soil profile, we analyzed samples 0-80 cm in depth at the Blodgett Experimental Forest in the central Sierra Nevada, CA. The warming treatment warmed the soil +4°C to 1 m depth while maintaining the natural temperature gradient. There were three control plots and three heated plots consisting of 3 m diameter circular plots each. Two concentric rings of heater cable at 1 and 2 m in diameter, 5 cm below the soil surface, to compensate for surface heat loss. Cables were installed similarly in control plots but was never connected to power. The amount of power supplied to the deep heaters was based on the temperature difference between paired control and heated thermistors at 75 and 100 cm depth and the surface heaters was based on the temperature difference of thermistors at 15 and 20 cm depth. Further details can be found in: Pries, C.E.H., Castanha, C., Porras, R.C. and Torn, M.S., 2017. The whole-soil carbon flux in response to warming. Science, 355(6332), pp.1420-1423.
Research sample	Soil samples from control and heated plots were collected at different depths to contrast the effects of soil warming through the soil profile.
Sampling strategy	Sample size is limited to the number of in-situ plots: control (n=3) and heated (n=3). We sampled one soil core from each plot using a 5-cm diameter AMS corer and extracted soils from the following depths: 0-10 cm, 10-30 cm, 30-45 cm, 45-60 cm, and 60-80 cm. Samples were homogenized in sterile bags and a subsample was immediate placed on dry ice for DNA extraction. The remaining sample was placed on “blue”ice (4 °C) and refrigerated in the laboratory for 2 days until the laboratory incubation experiment. Each depth yielded at least 50g soil.
Data collection	Data collected manually recorded in Excel and R data tables by Nicholas Dove.
Timing and spatial scale	One time sampling in June 2018 to observe microbial peak activity at end end of the rainy season. In-situ warming plots spans through a transect 100 m and soils were samples up to the depth of 80 cm.
Data exclusions	No data was excluded
Reproducibility	All measurements done here could only be performed one time. As we aimed at analyzing samples collected from the environment it would not be possible for us to be able to repeat the experiment and provide exactly the same conditions while doing so.
Randomization	Study has a block design and contrasts controls with heat treatment. Paired field experimental plots were set up identically and were randomly assigned +4 C heating or ambient (control) heating by randomly heating one of the paired plots. One sample at each depth was randomly collected within each plot. In the laboratory experiment, field-collected samples received either a cellulobiose addition, cellulobiose plus nutrient addition, or no addition (control) and were assigned heating corresponding to the experimental plot from which the samples were collected (14 C for heating and 10 C for ambient control).
Blinding	Blinding is not necessary for this study as we do not study human subjects.

Did the study involve field work? ☒ Yes ☐ No

Field work, collection and transport

Field conditions	Samples are collected at end end of the rainy season. Mean annual precipitation is 1774 mm, and mean annual air temperature is

	about 12.5 °C.
Location	The University of California Blodgett Experimental Forest is located in the foothills of the central Sierra Nevada near Georgetown, CA at 1370 m above sea level (38.886306; -120.647694)
Access & import/export	Permissions granted to LBNL Terrestrial Ecosystem Science Program (TES) Science Focus Area (SFA) 7/15/2013 by the University of California Berkeley
Disturbance	Soils that are destructively sampled were filled, no plant or animal life was disturbed during sampling

Reporting for specific materials, systems and methods

We require information from authors about some types of materials, experimental systems and methods used in many studies. Here, indicate whether each material, system or method listed is relevant to your study. If you are not sure if a list item applies to your research, read the appropriate section before selecting a response.

Materials & experimental systems

n/a	Involved in the study
☒	☐ Antibodies
☒	☐ Eukaryotic cell lines
☒	☐ Palaeontology and archaeology
☒	☐ Animals and other organisms
☒	☐ Human research participants
☒	☐ Clinical data
☒	☐ Dual use research of concern

Methods

n/a	Involved in the study
☒	☐ ChIP-seq
☒	☐ Flow cytometry
☒	☐ MRI-based neuroimaging
